# Parental Knowledge, Attitudes, and Utilization of Preventive Dentistry Modalities in Al Qassim, Saudi Arabia: A Cross-Sectional Study

**DOI:** 10.7759/cureus.70380

**Published:** 2024-09-28

**Authors:** Mohammed Ali Habibullah, Khalid S Almutairi, Rayan S Almutairi

**Affiliations:** 1 Preventive Dentistry, Qassim University, Buraydah, SAU; 2 General Dentistry, Qassim University, Buraydah, SAU

**Keywords:** dental health, oral health, parental knowledge, pediatric dental health, preventive dentistry

## Abstract

Background

Parents play a vital role in modeling good oral hygiene practices for their children; thus, their attitude, knowledge, and awareness toward preventive dentistry can be one of the major factors contributing to children’s oral health.

Objectives

The objective of this study is to determine the parental knowledge and attitudes toward the modalities of preventive dentistry for their children in Al Qassim, Saudi Arabia.

Materials and methods

A cross-sectional study was carried out using an online self-administered questionnaire designed for parents living in the Al Qassim region. The questionnaire included demographic data, parent knowledge and attitude toward preventive dentistry, and awareness of preventive dentistry procedures. The data collected were analyzed using RStudio.

Results

Data were collected from 392 Saudi parents (male: n = 190 (48.5%), female: n = 202 (51.5%)). The majority of participants (n = 336, 85.7%) considered the importance of primary teeth for general body health, while 320 participants (81.6%) believed that primary dentition influences permanent teeth. Awareness regarding special preventive measures like using toothpaste with fluorides comprised 87.5% (n = 343), and that of the benefits of early dental visits was 77.3% (n = 303). However, there was marked unawareness and use of preventive procedures such as serial extraction (n = 226, 57.7% of respondents unaware) and silver diamine fluoride (n = 276, 70.4% unaware). The higher levels of education and income were positively related to better knowledge and attitudes about preventive dentistry.

Conclusions

The current paper highlights the importance of educational interventions for enhancing parental knowledge and awareness about preventive dentistry. Community-based educational programs and increased availability of preventive dentistry services might be helpful in eliminating the knowledge gap among parents about good oral health practice.

## Introduction

Early diagnosis and treatment of oral health conditions can prevent many complications in later life. Primary prevention aims at the initial stages, whereas secondary prevention aims to stop disease progression [1]. Preventing dental plaque at its earliest stage is most effective when it occurs at its initial stage. Proper oral hygiene measures, decreasing dietary sugar exposure, use of mouthwashes, and eating fibrous foods can go a long way in preventing the accumulation of dental plaque. In addition to population-based prevention, routine dental checkups spaced at six-month intervals are the most effective means of preventing dental decay [2].

A child’s oral health greatly depends on the parental choices regarding dental therapy [3]. It is important to note here that oral health can also be achieved through preventive means with less time and cost to the patient. Fluorides, both topical and systemic, can be used to prevent dental disease in children [4]. Remineralizing dentifrices/chewing gums, pit and fissure sealants, fluoride varnish, mouth guards to prevent contact sports injuries, and interceptive orthodontics for malocclusion prevention all aid in optimum oral health for the child [5]. Professional guidance is required for these procedures. Minor changes can be corrected by dental appliances that aid in guided jaw growth but may have an adverse effect at a later stage [6]. Therefore, parents' preventive oral health behaviors will likely influence their children’s behavior when they adopt preventive oral health practices as they grow [7]. Parental role is the most important factor in maintaining good oral health in preschool children [8,9]. Parents should become familiar with preventive dentistry procedures to take advantage of available dental services. Thus, parents who take preventive oral health seriously can have a tremendous impact on their children’s oral health for the rest of their lives.

However, parental knowledge and attitude toward preventive dentistry were rarely investigated in the Al Qassim region of Saudi Arabia. This research gap makes evident the need for an in-depth understanding of the perception and preventive dental practices of the involved parent, as the involvement is pivotal for shaping oral health behaviors and outcomes in children. Therefore, the current study aimed to identify the current level of knowledge and attitude of parents about the different modalities of preventive dentistry for children in Al Qassim, Saudi Arabia. When we consider studies in Saudi Arabia, there has been a similar study conducted in Riyadh. Riyadh, the capital of Saudi Arabia, is a well-developed urban metropolis, and this study consisted of people from an urban population [2]. Our sample is derived from a suburban/rural population that provides a different perspective on the same problem. This research gap makes evident the need for an in-depth understanding of the perception and preventive dental practices of the involved parent, as the involvement is pivotal for shaping oral health behaviors and outcomes in children. Therefore, the current study aimed to identify the current level of knowledge and attitude of parents about the different modalities of preventive dentistry for children in Al Qassim, Saudi Arabia.

## Materials and methods

Study design and setting

A cross-sectional study was conducted in the Al Qassim region of Saudi Arabia. The study design involved the distribution of a self-administered questionnaire online to parents. Ethical approval was obtained from the institutional review board of Qassim University (approval number 23-67-20).

Sample size calculation and sampling technique

With a sampling frame of 1,016,756 [10] and a CI of 95% with a margin of error of 5%, and assuming a response distribution of 50%, the sample size was determined to be 385 using an online calculator (Raosoft sample size calculator) [11]. The sample size was determined using non-probability sampling. Inclusion criteria comprised Saudi citizens residing in the Qassim region with at least one child and providing informed consent. Exclusion criteria included Saudi citizens residing outside the geographical limits of the Al Qassim Region and parents refusing to participate or failing to provide consent.

Data collection and the study tool

Data collection involved an online questionnaire consisting of three parts: the first part focused on collecting sociodemographic details of respondents, while the second part comprised close-ended questions aimed at determining parental attitudes regarding preventive dentistry modalities for their children. The third part included items that measure parental awareness and utilization of preventive dentistry. The second and third parts were adapted from a previously published article [2]. All data were stored anonymously, ensuring no personal identification of respondents except for age and gender. Access to the data was restricted to the principal and co-investigator to uphold ethical standards.

Validation of the questionnaire

The validation of subdomains was implemented using Cronbach’s alpha testing. Results showed good internal consistency parameters for parent knowledge and attitudes (9 items, alpha = 0.809) and awareness regarding preventive dentistry (9 items, alpha = 0.713).

Calculation of the attitude score

To assess the levels of parents’ attitudes, we assigned 1 to the “Agree” responses and 0 to other responses for the nine items of attitudes. Subsequently, we created a score variable by summing up the values of attitudes items. Therefore, the overall score ranged between 0 and 9.

Statistical analysis

Statistical analysis was performed using RStudio (R version 4.3.1). We used frequencies and percentages to express categorical variables, whereas numerical variables were presented as mean ± SD. The differences in parents’ attitudes and awareness in terms of demographic characteristics were assessed using a Fisher’s exact test. The differences in the scores of attitudes across different demographic groups were evaluated using a Kruskal-Wallis rank sum test. A multivariable linear regression model was constructed to assess the independent associations with the attitudes score, and the results were expressed as beta coefficients and 95% CIs. Statistical significance was defined at p < 0.05.

## Results

Demographic characteristics

Data were collected from 392 parents in the current study. The sample for this study was derived predominantly from a suburban and, to a lesser extent, from a rural population. The age group with the highest frequency was 40-50 years, constituting 36.2% of the total participants. Regarding educational status, participants with a bachelor’s degree were the most prevalent, comprising 42.3% of the sample. In terms of monthly income, the majority of participants fell into the income bracket of 10,000 to 20,000 SAR, representing 31.1% of the respondents (Table 1).

**Table 1:** Demographic characteristics

Characteristic	N (%)
Age (year)	
23-30	124 (31.6%)
30-40	100 (25.5%)
40-50	142 (36.2%)
>50	26 (6.6%)
Educational status	
No schooling	14 (3.6%)
Up to secondary	33 (8.4%)
Bachelor	166 (42.3%)
Master	150 (38.3%)
PhD	29 (7.4%)
Monthly income (SAR)	
<4,000	95 (24.2%)
4,000-10,000	76 (19.4%)
10,000-20,000	122 (31.1%)
>20,000	99 (25.3%)

Parental responses to attitudes items

**Figure 1:**
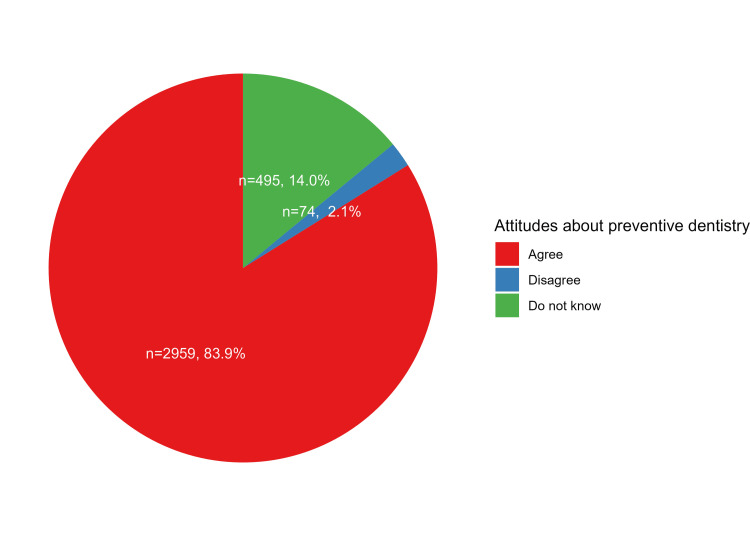
Parental knowledge and attitudes regarding preventive dentistry (responses of 392 participants to nine questions)

In general, the majority expressed positive attitudes toward preventive dentistry procedures, as indicated by their agreement to the statements of attitudes by 83.9% of the sample (Figure 1). In particular, the majority of parents agreed with the importance of primary teeth for their child’s health (85.7%), recognized the impact of primary teeth on permanent teeth (81.6%), acknowledged the link between oral health and general health (85.2%), understood that using fluoridated toothpaste helps prevent tooth decay (87.5%), believed that parents play a significant role in shaping a child's dental attitude (92.3%), recognized the importance of early dental visits (77.3%), acknowledged that the correct method of cleaning teeth can prevent dental decay (94.9%), understood the potential adverse effects of prolonged pacifier use on normal dental development (76.5%), and believed that malocclusion can be prevented (73.7%, Table 2). Of note, there were significant differences in all items of attitudes in terms of parents’ age categories, educational levels, and monthly income levels (Table 2).

**Table 2:** Parental responses to attitudes items regarding preventive dentistry Fisher’s exact test

Characteristic	Overall	Age	Educational level	Monthly income
	n = 392	p-value	p-value	p-value
Primary teeth are important for a child’s health.		<0.001	<0.001	<0.001
Disagree	6 (1.5%)			
Agree	336 (85.7%)			
Do not know	50 (12.8%)			
Primary teeth will affect a child’s permanent teeth.		<0.001	<0.001	<0.001
Disagree	9 (2.3%)			
Agree	320 (81.6%)			
Do not know	63 (16.1%)			
Oral health affects general health.		<0.001	<0.001	<0.001
Disagree	11 (2.8%)			
Agree	334 (85.2%)			
Do not know	47 (12.0%)			
Using fluoridated toothpaste helps to prevent tooth decay.		<0.001	<0.001	<0.001
Disagree	4 (1.0%)			
Agree	343 (87.5%)			
Do not know	45 (11.5%)			
Parents have an important role in developing a child’s dental attitude.		<0.001	0.048	0.002
Disagree	7 (1.8%)			
Agree	362 (92.3%)			
Do not know	23 (5.9%)			
It is important to visit the dentist as early as possible.		<0.001	<0.001	<0.001
Disagree	17 (4.3%)			
Agree	303 (77.3%)			
Do not know	72 (18.4%)			
Correct method of cleaning teeth can prevent dental decay.		0.017	<0.001	0.161
Disagree	3 (0.8%)			
Agree	372 (94.9%)			
Do not know	17 (4.3%)			
Prolonged use of pacifiers can affect the normal development of a child’s teeth.		<0.001	<0.001	<0.001
Disagree	6 (1.5%)			
Agree	300 (76.5%)			
Do not know	86 (21.9%)			
Malocclusion can be preventable.		<0.001	<0.001	<0.001
Disagree	11 (2.8%)			
Agree	289 (73.7%)			
Do not know	92 (23.5%)			

Focusing on the numerical attitudes score, there were statistically significant differences in the scores based on parents’ age (p < 0.001), educational status (p < 0.001), and monthly income (p < 0.001). On the multivariable regression analysis, parents aged 30 to 40 years (beta = 1.52, 95% CI: 0.92-2.12, p < 0.001), 40-50 years (beta = 1.45, 95% CI: 0.79-2.12, p < 0.001), and over 50 years (beta = 1.46, 95% CI: 0.40-2.51, p = 0.007) demonstrated higher levels of attitudes compared to those aged 23-30 years. Regarding educational attainment, parents with a PhD degree exhibited significantly positive attitudes (beta = 1.35, 95% CI: 0.05-2.64, p = 0.043) compared to those with no schooling. Moreover, higher monthly income levels, including 4,000-10,000 SAR (beta = 0.87, 95% CI: 0.27-1.48, p = 0.005), 10,000-20,000 SAR (beta = 1.00, 95% CI: 0.33-1.67, p = 0.004), and >20,000 SAR (beta = 0.94, 95% CI: 0.17-1.70, p = 0.017), were associated with positive compared to income below 4,000 SAR (Table 3). In other words, age greater than 30, higher educational attainment, and higher income (<4,000 SAR) proved to be accurate predictors for good attitudes toward preventive dental care in our study.

**Table 3:** Results of the univariable and multivariable analyses for the factors associated with parental attitudes regarding preventive dentistry * Kruskal-Wallis rank sum test

Characteristic	Inferential analysis*			Multivariable regression		
	Attitude score (mean ± SD)	Range (min-max)	p-value	Beta	95% CI	p-value
Age (year)			<0.001			
23-30	5.89 ± 2.52	1-9		Reference	Reference	
30-40	8.14 ± 1.72	1-9		1.52	0.92, 2.12	<0.001
40-50	8.41 ± 1.41	2-9		1.45	0.79, 2.12	<0.001
>50	8.50 ± 1.14	5-9		1.46	0.40, 2.51	0.007
Educational status			<0.001			
No schooling	6.71 ± 2.87	2-9		Reference	Reference	
Up to secondary	6.27 ± 2.92	1-9		0.29	-0.89, 1.46	0.633
Bachelor	6.77 ± 2.35	1-9		0.47	-0.57, 1.50	0.376
Master	8.53 ± 1.23	1-9		1	-0.07, 2.07	0.069
PhD	8.83 ± 0.76	5-9		1.35	0.05, 2.64	0.043
Monthly income (SAR)			<0.001			
<4,000	5.83 ± 2.62	1-9		Reference	Reference	
4,000-10,000	7.41 ± 2.08	2-9		0.87	0.27, 1.48	0.005
10,000-20,000	8.26 ± 1.69	2-9		1	0.33, 1.67	0.004
>20,000	8.42 ± 1.25	1-9		0.94	0.17, 1.70	0.017

Awareness and utilization regarding preventive dentistry

**Figure 2:**
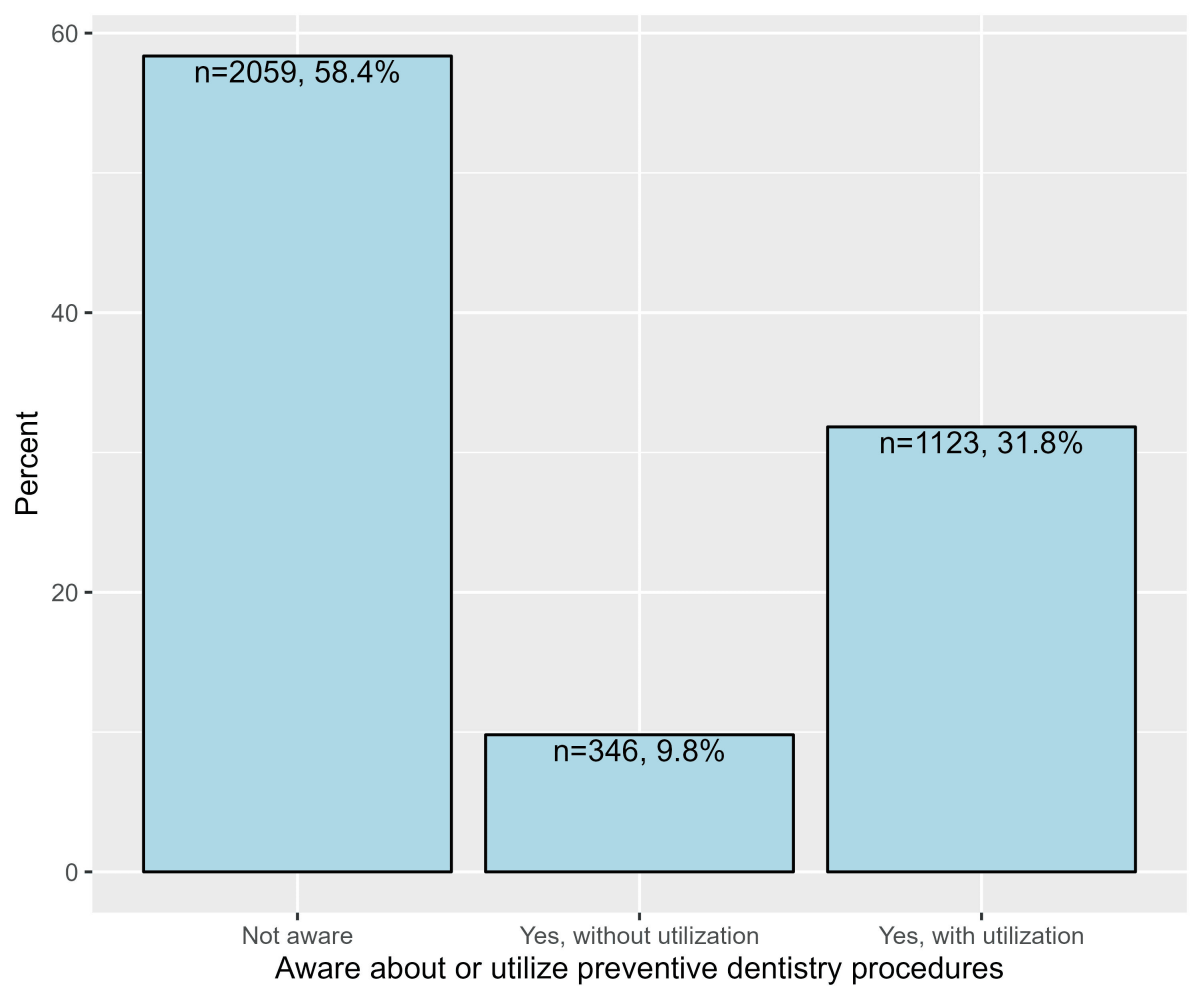
Proportions of parental awareness and utilization of preventive dentistry procedures (responses of 392 participants to nine questions)

Generally, approximately one-third of parents utilized preventive dentistry procedures (31.8%), whereas 9.8% of them were aware but did not utilize these procedures. On the other hand, more than half of parents (58.4%) were not aware of the procedures (Figure 2). Additionally, more than half of parents were not aware of or utilized serial extraction (57.7%), topical fluoride application (59.9%), pit and fissure sealants (68.4%), fluoride varnish (62.2%), silver diamine fluoride (SDF; 70.4%), and space maintainers (71.9%). However, considerable proportions of parents had utilized mouth guards (39.3%), dental floss (39.5%), and fluoride mouth rinses (45.7%, Table 4).

**Table 4:** Parental responses to awareness and utilization items regarding preventive dentistry Fisher’s exact test

Characteristic	Overall, n = 392	p-value		
		Age	Educational status	Monthly income
Serial extraction		0.015	0.01	0.006
Not aware	226 (57.7%)			
Yes, without utilization	36 (9.2%)			
Yes, with utilization	130 (33.2%)			
Topical fluoride application		0.274	0.009	0.051
Not aware	235 (59.9%)			
Yes, without utilization	33 (8.4%)			
Yes, with utilization	124 (31.6%)			
Mouth guards		0.015	<0.001	<0.001
Not aware	178 (45.4%)			
Yes, without utilization	60 (15.3%)			
Yes, with utilization	154 (39.3%)			
Pit and fissure sealants		0.112	0.003	0.016
Not aware	268 (68.4%)			
Yes, without utilization	33 (8.4%)			
Yes, with utilization	91 (23.2%)			
Fluoride varnish		0.049	0.003	0.003
Not aware	244 (62.2%)			
Yes, without utilization	31 (7.9%)			
Yes, with utilization	117 (29.8%)			
Dental floss		0.002	<0.001	<0.001
Not aware	183 (46.7%)			
Yes, without utilization	54 (13.8%)			
Yes, with utilization	155 (39.5%)			
Fluoride mouth rinses		0.122	<0.001	<0.001
Not aware	167 (42.6%)			
Yes, without utilization	46 (11.7%)			
Yes, with utilization	179 (45.7%)			
Silver diamine fluoride		0.864	0.053	0.164
Not aware	276 (70.4%)			
Yes, without utilization	21 (5.4%)			
Yes, with utilization	95 (24.2%)			
Space maintainer		0.133	0.002	0.023
Not aware	282 (71.9%)			
Yes, without utilization	32 (8.2%)			
Yes, with utilization	78 (19.9%)			

In terms of the differences between demographic categories, awareness and utilization levels differed significantly based on age in terms of serial extraction (p = 0.015), mouth guards (p = 0.015), fluoride varnish (p = 0.049), and dental floss (p = 0.002). Based on the education levels, significant differences were noted for the awareness and utilization of serial extraction (p = 0.010), topical fluoride application (p = 0.009), mouth guards (p < 0.001), pit and fissure sealants (p = 0.003), fluoride varnish (p = 0.003), dental floss (p < 0.001), fluoride mouth rinses (p < 0.001), and space maintainers (p = 0.002). For the monthly income, there were significant differences based on the awareness and attitudes regarding serial extraction (p = 0.006), mouth guards (p < 0.001), pit and fissure sealants (p = 0.016), fluoride varnish (p = 0.003), dental floss (p < 0.001), fluoride mouth rinses (p < 0.001), and space maintainers (p = 0.023, Table 4).

## Discussion

Given the critical role parents play in shaping their children’s oral health behaviors, it is essential to understand their level of awareness and attitudes toward preventive dental practices. The study results revealed that most participants recognized the importance of primary teeth for overall health and understood their impact on permanent teeth. Awareness of specific preventive measures, such as using fluoridated toothpaste and early dental visits, was high among parents. However, significant gaps were identified in the awareness and utilization of certain preventive procedures, particularly serial extraction and SDF. The study also found that higher education and income levels were associated with better knowledge and attitudes toward preventive dentistry.

Based on previous research, a substantial majority of Malaysian parents (82.3%) recognize the importance of primary teeth, whereas we found that the percentage of respondents to our study was slightly higher (85.7%), which is in agreement with those from other studies [12].

The results of the study indicate that 85.2% of the participants understood that oral health could have an impact on general health. This matched previous research in Manitoba that had shown that 87.5% of respondents acknowledged that dental diseases could have an impact on general health [13].

Severe crowding caused by tooth size and arch length deficiency can be managed early with serial extractions in the mixed dentition or later by removing premolars in the permanent dentition [14]. Awareness regarding serial extraction and utilization patterns showed 57.7% were not aware, 9.2% of parents were aware but not utilizing, and 33.2% were aware and utilizing these preventive modalities.

The awareness and utilization rates of topical fluoride application in our study were higher compared to Almalki et al. [2] study in Riyadh. In our study, 59.9% were unaware, 8.4% were aware but did not utilize, and 31.6% were aware and utilized. In contrast, the Riyadh study reported lower rates with 43.3% awareness and 17.7% utilization.

Our study on pit and fissure sealants revealed that 68.4% of people were unaware, 8.4% were aware but not using them, and 23.2% were aware and using them. Compared to Almalki et al. [2], the study revealed 58.0% awareness and 13.3% utilization. These findings suggest that there is still a significant lack of awareness and utilization of topical fluoride application and pit and fissure sealants in both studies. This indicates a need for increased education and promotion of these dental preventive measures to improve oral health outcomes in both populations.

In our study, 42.6% of respondents are not aware of fluoride mouth rinses, 11.7% are aware but are not using them, and 45.7% are aware of and using them. As per Almalki et al. [2], 54.3% of participants are aware, while 4.0% are using fluoride mouth rinses.

We found that 39.5% of parents reported that their children used dental floss, which is significantly higher than the 14.6% reported in Mumbai [15].

The results of our study showed that 71.9% of respondents were unaware of space maintainers. In comparison, Alduraihim et al. [16] study conducted in Al-Kharj documented that 82.1% of parents were unaware of space maintainers as well.

As a result of the strong correlation between education and moderate income, but not for age, the knowledge and application of space maintainers appear to be more influenced by socioeconomic factors than by parents’ age.

In this study, the awareness and use of mouth guards. Approximately 45.4% of the population was unaware of mouth guards. Moreover, 15.3% were aware but did not use mouth guards. A total of 39.3% were both aware and used mouth guards. In accordance with the study by Soares et al. [17], awareness among parents revealed that initially, 54.9% did not understand the meaning of a mouth guard. However, after explanations, 57.4% recognized the use of a mouth guard. These findings call for a targeted intervention approach to increasing awareness regarding mouth guards. These could possibly include the use of educational videos/posters in schools and the training and utilization of teachers and physical education staff to target the children, especially those engaging in athletics and contact sports.

According to the highly significant p-value, education has an important impact on awareness of mouth guards and their use, which greatly protects children’s teeth during sports. A higher income level generally provides greater access to health services, including preventative dental care products such as mouth guards. As a result of the significant p-value for age, younger parents are more likely to be aware of and utilize mouth guards. In part, this could be due to the fact that younger parents are more aware of the importance of protective gear or are more involved in active lifestyles, including sports.

In terms of prevention of dental caries, SDF has shown significant success, especially in cases of early childhood caries [18,19], root caries prevention and arrest [20], and dentin sensitivity [21]. This study shows that 70.4% of the population is unaware of SDF, while 5.4% are aware but are not utilizing it, and 24.2% are aware and utilizing it.

This p-value of education status is within the conventional threshold of significance of 0.05, suggesting that education is borderline significant in relation to SDF awareness and utilization. In light of this, parents with higher levels of education may have an increased chance of becoming aware of and using SDF. As evidenced by the lack of significant influence of income levels, parents are not strongly influenced by financial resources in the decision to use SDF for their children. The high p-value of 0.864 shows that the age of the parents does not affect the awareness or use of SDF.

Our study has focused on a population that has not been the subject of earlier studies. We have used a validated questionnaire with a sufficiently large sample size drawn from a suburban/rural population to arrive at conclusions that will assist in targeted interventions for this population group. However, the present study is not without limitations. The cross-sectional nature of the study limits the ability to confirm causal relationships between knowledge and attitudes and preventive dental practices. The use of a self-administered online survey imposes another limitation, where self-reporting bias may be apparent since parents might have overestimated their knowledge and attitudes toward preventive dentistry. Furthermore, the study sample was restricted to Saudi parents residing in the Al-Qassim region, which might induce limited generalizability to other regions in the Kingdom. Finally, the non-probability sampling technique might not have represented the broader population, which might have influenced the interpretations of the findings. Future studies might employ longitudinal designs, recruit parents from multiple regions and provinces, and use randomized sampling methods to eventually obtain validated results.

## Conclusions

Older and more educated parents, particularly those with higher incomes, demonstrated a more positive attitude toward preventive dental care. Most respondents recognized the importance of primary teeth, as well as the link between oral health and general health. However, there was a significant lack of awareness and utilization of treatments such as serial extractions and fluoride treatments. The analysis highlighted that education and income levels were critical in influencing these attitudes, suggesting the need for targeted education programs to enhance preventive dentistry awareness and practices across all socioeconomic groups. It is possible that this will improve the oral health outcomes of children in the region.

## Appendices

**Table 5:** Questionnaire used in the current study

Domain	Variable	Responses				
Demographic characteristics	Age	23-30	30-40	40-50	>50	
	Educational status	No schooling	Up to secondary	Bachelor	Master	PhD
	Monthly income	<4,000	4,000-10,000	10,000-20,000	>20,000	
Parental knowledge and attitudes regarding preventive dentistry	Primary teeth are important for child’s health	Disagree	Agree	Do not know		
	Primary teeth will affect child’s permanent teeth	Disagree	Agree	Do not know		
	Oral health affects general health	Disagree	Agree	Do not know		
	Using fluoridated toothpaste helps to prevent tooth decay	Disagree	Agree	Do not know		
	Parents have an important role in developing a child’s dental attitude	Disagree	Agree	Do not know		
	It is important to visit the dentist as early as possible	Disagree	Agree	Do not know		
	Correct method of cleaning teeth can prevent dental decay	Disagree	Agree	Do not know		
	Prolonged use of pacifier can affect the normal development of child's teeth	Disagree	Agree	Do not know		
	Malocclusion can be preventable	Disagree	Agree	Do not know		
Awareness of preventive dentistry procedures	Serial extraction	Not aware	Yes, without utilization	Yes, with utilization		
	Topical fluoride application	Not aware	Yes, without utilization	Yes, with utilization		
	Mouth guards	Not aware	Yes, without utilization	Yes, with utilization		
	Pit and fissure sealants	Not aware	Yes, without utilization	Yes, with utilization		
	Fluoride varnish	Not aware	Yes, without utilization	Yes, with utilization		
	Dental floss	Not aware	Yes, without utilization	Yes, with utilization		
	Fluoride mouth rinses	Not aware	Yes, without utilization	Yes, with utilization		
	Silver diamine fluoride	Not aware	Yes, without utilization	Yes, with utilization		
	Space maintainer	Not aware	Yes, without utilization	Yes, with utilization		
